# Circulating Placental Extracellular Vesicles and Their Potential Roles During Pregnancy

**DOI:** 10.31486/toj.20.0049

**Published:** 2020

**Authors:** Angela Nakahara, Soumyalekshmi Nair, Valeska Ormazabal, Omar Elfeky, Cathryn E. Garvey, Sherri Longo, Carlos Salomon

**Affiliations:** ^1^ Exosome Biology Laboratory, Centre for Clinical Diagnostics, University of Queensland Centre for Clinical Research, Royal Brisbane and Women's Hospital, The University of Queensland, Herston, Queensland, Australia; ^2^ Department of Obstetrics and Gynecology, Maternal-Fetal Medicine, Ochsner Clinic Foundation, New Orleans, LA; ^3^ The University of Queensland Faculty of Medicine, Ochsner Clinical School, New Orleans, LA; ^4^ Department of Pharmacology, Faculty of Biological Sciences, University of Concepción, Concepción, Chile

**Keywords:** *Exosomes*, *placenta*, *pregnancy*

## Abstract

**Background:** Numerous changes in maternal physiology occur during pregnancy that are critical in controlling and maintaining the maternal metabolic adaptations and fetal development. The placenta is the key source through which the fetus receives nutrients, blood, and oxygen for growth. The human placenta releases several molecules into maternal circulation that include hormones, proteins, RNA, and DNA throughout the course of pregnancy. Additionally, extracellular vesicles (EVs) originating from the placenta have been found in the maternal circulation.

**Methods:** In this review, we discuss the role of EVs in maternal-fetal communication during pregnancy.

**Results:** EVs originating from the placenta can be divided into 3 categories based on their size and/or origin: exosomes (50 to 150 nm), microvesicles (nm to several μm), and apoptotic bodies or syncytial nuclear aggregates (>1 μm). The cellular microenvironment—such as oxygen tension and glucose concentration—have been found to control EV release from the placenta and their bioactivity on target cells. Furthermore, maternal EVs can stimulate cytokine release from endothelial cells and are involved in several physiologic and pathologic events in pregnancy.

**Conclusion:** Exosomes provide a way to identify the function and metabolic state of cell origin through their ability to reflect the microenvironment that they are released from. Further understanding of how EVs regulate key events in pregnancy may help elucidate how maternal-fetal communication is established in both normal and pathologic conditions.

## INTRODUCTION

The human placenta develops from the implantation of the blastocyst into the uterine wall and becomes a critical transient organ that is unique to pregnancy.^1^ The placenta provides nutrition, gas exchange, waste removal, and immune support for the fetus and also mediates molecular exchange between the maternal and fetal systems.^2^

The placenta is the home of trophoblast cells that can be subcategorized as cytotrophoblasts, extravillous trophoblasts, or syncytiotrophoblasts. The placenta releases many different molecules that can alter maternal physiology to accommodate fetal requirements during gestation. The placenta may also influence the physiology of the mother via extracellular vesicles (EVs).^3-5^

EVs are increasingly released into the maternal circulation as pregnancy progresses, in both healthy and pathologic pregnancies (ie, pregnancies complicated by gestational diabetes mellitus [GDM]) or preeclampsia [PE]) (Figure).^4-6^ EVs encapsulate a diverse cargo of proteins, lipids, and nucleic acids that are released into the maternal circulation and are subsequently taken up by cells of the maternal immune and vascular systems, thereby modulating the overall maternal physiologic system to adapt to pregnancy-induced changes.^4^ In complicated pregnancies, this mode of cell signaling plays a role in the manifestation of physical symptoms of disease states, as the release of the EVs is dependent on the microenvironment to which they are exposed.^7^

**Figure.**
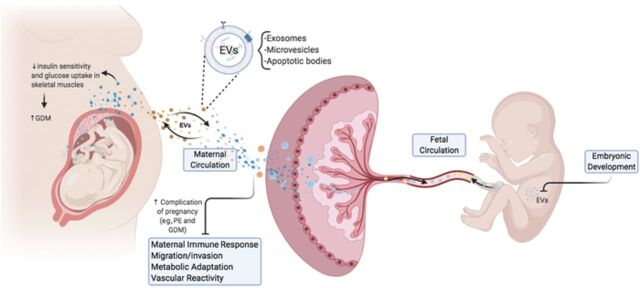
**Extracellular vesicle (EV) trafficking during pregnancy. EVs—including small EVs such as exosomes and large EVs such as microvesicles and apoptotic bodies—are released from the human placenta into maternal and fetal circulation during pregnancy. Placental EVs present in maternal circulation can interact with maternal tissues and regulate several biologic functions, including maternal immune response, migration/invasion, metabolic adaptation to pregnancy, and vascular reactivity. EVs present in fetal circulation are associated with fetal development.** GDM, gestational diabetes mellitus; PE, preeclampsia.

Several recent (2014 through 2020) reviews on EVs during pregnancy have been published,^8-11^ but this review focuses on EVs during gestation, with emphasis on the trafficking of placental vesicles into maternal circulation to regulate immune and metabolic adaptations in pregnancy.

## GENERAL CHARACTERISTICS OF EXTRACELLULAR VESICLES

Investigation of the potential role of EVs in intercellular communication has been an area of growing research.^12^ EVs are lipid bilayer structures measuring 50 nm to several μm that are released from a multitude of different cell types, including trophoblasts,^13^ erythrocytes,^14^ and endothelial cells,^15^ into the extracellular environment. Within their lipid bilayer membrane, EVs contain RNA, DNA, and proteins, which are hypothesized to modulate the bioactivity of specifically targeted cells.^16^ Studies published in 2014 and 2018 have demonstrated that EVs may affect cellular adaptations to physiologic changes during gestation, including immune responses, migration, and invasion of placental cells.^17,18^

The different types of EVs—exosomes, microvesicles, and apoptotic bodies—can be categorized on the basis of their size, surface markers, and content.^12^ Exosomes are a specific type of EV (50 to 150 nm) that arise from the inward budding of the plasma membrane, forming multivesicular bodies that internalize to form intraluminal vesicles and are then released into the extracellular environment as exosomes. As a result of their mode of formation, exosomes are enriched with intracellular proteins such as CD63, CD9, and CD81. Exosomes are distinguished from other EV cells by their distinct cup-shaped morphology and a buoyant density in a sucrose gradient, ranging from 1.13 to 1.19 g/mL. Compared to exosomes, microvesicles are different in size and originate in a different manner. Microvesicles can be as small as exosomes or as large as a few μm in size and are derived from plasma membrane secretion through direct budding of the membrane itself in response to cellular activation or in response to stress.^19^ Similarly, apoptotic bodies differ from exosomes in size (>1 μm) and through their formation via membrane blebbing, membrane protrusion, and formation of distinct bodies.

Regardless of their origin and size, EVs play important roles in cell communication and act as key regulators in disease states and healthy conditions. In the context of pregnancy, the placenta releases a wide range of EVs that are identified in the maternal circulation in both normal pregnancies and pregnancies with complications.

## PLACENTAL VESICLES IN MATERNAL CIRCULATION

Exosomes are released from different organs, including the placenta, in both normal and pathologic conditions.^5^ Placental exosomes are hypothesized to regulate the establishment of maternal-fetal circulation via remodeling of spiral arteries. Upon implantation, the blastocyst adheres to the endothelium of the placenta, and the trophoblastic cells of the fetus differentiate into an inner cytotrophoblast and an outer syncytiotrophoblast layer. As the blastocyst continues to grow, the cytotrophoblast layer forms a layer of multinucleated syncytiotrophoblasts. Within this layer, the cytotrophoblast releases proteolytic enzymes, while the syncytiotrophoblast layer extends finger-like projections into the endometrium and replaces the endothelial cells of the uterine spiral arteries, establishing a connection between the growing embryo and maternal blood. This normal physiologic process is thought to be mediated at least in part by cell-to-cell communication via EVs and is complete by 10 weeks of gestation.

Evidence of EV release by extravillous trophoblasts has been demonstrated through the detection of soluble proteins such as human leukocyte antigen G (HLA-G).^20^ Whereas HLA-G is expressed only in extravillous trophoblasts, HLA-G^+^-EVs have been detected in pregnancy.^21^ An analysis of EVs released from extravillous trophoblasts using Swan71 cell line isolation showed that the EVs release exosomes.^22^ The findings of this study strongly indicate that extravillous trophoblasts not only release microvesicles but specifically exosomes.

The amount and type of EVs released are affected by the microenvironment.^23,24^ In a comparison of pregnant and nonpregnant women, the concentration of exosomes was approximately 50-fold greater in pregnant women.^25^ The increased concentration may be attributable in part to the contribution of placental-specific exosomes that have been identified in maternal circulation as early as approximately 6 weeks of gestational age.^9^ Specifically, placental-alkaline phosphatase-positive (PLAP^+^)-EVs were detected in the maternal circulation throughout pregnancy.^5^ The PLAP^+^-EVs can be isolated from the plasma of pregnant women and increase during the first 12 weeks of gestation.^5^ The increased presence of these EVs may be because of the changes and adaptations in maternal physiology.

Changes in the cellular microenvironment, such as hypoxia or increased glucose concentration, also affect EV release. These different microenvironments can also affect vesicle content and the bioactivity of target cells.^23,26^ In pregnancies with increased stress, such as those complicated by GDM^6^ and PE,^27^ more EVs are released. For example, the circulation in women affected by PE contains approximately 40% greater number of EVs compared to healthy, pregnant women.^28^ Furthermore, studies have demonstrated that changes in the microenvironment can alter the secretion of EVs from cytotrophoblast cells and in turn affect vesicle content and bioactivity.^23,24^ Exosomes released from the placenta have been shown to decrease insulin sensitivity and glucose uptake in skeletal muscles, contributing to the pathophysiology of GDM.^29^ Also, exosomes released from other types of cells can affect placental function and are involved in regulating the physiologic and pathologic mechanisms in pregnancy. For example, exosomes released from adipocytes can mediate changes in placental metabolic status and contribute to GDM.^30^ Hence, the cross-communication between different organs and the placenta via exosomes is crucial in mediating the maternal metabolic changes in pregnancy.^31^

As previously stated, the role of exosomes in the remodeling of spiral arteries from a high-resistance, low-capacitance system to a low-resistance, high-capacitance system has been studied.^13,26,27^ Furthermore, studies have found that states of abnormal placentation, such as PE, have increased concentrations of circulating placental-derived EVs.^27,28,32^ All of these findings suggest that EVs have a significant role during pregnancy. The Table summarizes published studies characterizing EVs during gestation.^5,6,14,25,28,33-43^ Future studies are required to develop a proper understanding of the mechanisms of exosome release and their effect on the target cells, which could give insight into their role as markers for the diagnosis of pregnancy complications.

**Table.** Studies of Extracellular Vesicles in Maternal and Fetal Circulation

	Study	Extracellular Vesicle	Sample Collection Type	Gestational Age, weeks	Isolation Method	Pregnancy	Biological Process Analyzed
Maternal circulation	Sabapatha et al, 2006^34^	Exosomes	Plasma	28-30	Immunomagnetic isolation	Normal	Maternal immune response
	Luo et al, 2009^35^	Exosomes	Plasma	7-11 36-38	Centrifugation	Normal	miRNA analysis
	Sarker et al, 2014^5^	Exosomes	Plasma	6-12	Centrifugation and density gradient	Normal	Placental exosomes increase from 6-12 weeks
	Salomon et al, 2014^25^	Exosomes	Plasma	6-12 22-28 32-36	Centrifugation and density gradient	Normal	Exosomes increase across gestation
	Pillay et al, 2016^36^	Exosomes	Plasma	>30	Centrifugation and density gradient	PE	High levels of placental exosomes in PE
	Salomon et al, 2016^6^	Exosomes	Plasma	11-14 22-28 32-36	Centrifugation and density gradient	GDM	High levels of placental exosomes in GDM
	Ratajczak et al, 2013^38^	Microvesicles	Plasma	N/A	N/A	Normal	Angiogenesis
	da Silva Nardi et al, 2016^37^	Microvesicles	Plasma	12-18	Commercial kit	Normal	Immunologic role
	Goswami et al, 2006^28^	STMB	Plasma	>34	Centrifugation	PE, IUGR	High levels of placental STMB
	Chen et al, 2012^39^	STMB	Plasma	>34	Centrifugation	PE	High levels of placental STMB
	Dragovic et al, 2013^14^	STMB	Plasma	>37	Centrifugation	PE	High level of EVs in late-onset PE
	Moro et al, 2016^40^	Microparticles	Plasma	>37	Centrifugation	HIV infection	Immunologic role
	Lok et al, 2008^41^	Microparticles	Plasma	12-38	Centrifugation	PE	High levels of placental microparticles
Fetal circulation	Li et al, 2013^42^	MSC-derived exosomes	Umbilical vein blood	At delivery	Centrifugation and filtration	Normal	Cell therapy
	Zhou et al, 2013^43^	MSC-derived exosomes	Umbilical vein blood	At delivery	Centrifugation and filtration	Normal	Cell therapy
	Jia et al, 2015^33^	MSC-derived exosomes	Umbilical vein blood	At delivery	Centrifugation	PE	Proteomic analysis

GDM, gestational diabetes mellitus; HIV, human immunodeficiency virus; IUGR, intrauterine growth restriction; miRNA, micro-RNA; MSC, mesenchymal stem cells; N/A, not applicable; PE, preeclampsia; STMB, syncytiotrophoblast microvesicles.

## POTENTIAL ROLES OF EXTRACELLULAR VESICLES DURING GESTATION

In normal physiology, EVs from the placenta balance both immunosuppressive and proinflammatory cytokines to support fetal establishment and curtail rejection. Because a fetus is antigenically unique from the mother, maternal immune responses against the growing organism must at least in some part be avoided by inhibiting maternal T lymphocyte (T cell) and natural killer (NK) cell activation.^44^ As stated previously, exosomes correlationally increase with increasing gestational age,^5,6^ and several studies have explored the potential role of placental-derived exosomes in regulating maternal immune response during pregnancy.^20,45-48^

More specifically, immune adaptations in pregnancy are believed to be largely attributable to the expression and production of different cytokines that are thought to be regulated by EVs.^18,49^ Throughout pregnancy, proinflammatory and anti-inflammatory stages allow for the development, maturation, and parturition of the fetus.^49^ Placental EVs are believed to have a role in modulating these proinflammation and anti-inflammation states by modulating cytokine release.^6,20^ A method through which this regulation occurs could be via the expression of UL16 binding protein 1-5 (ULBP1-5) and major histocompatibility complex (MHC) class I chain-related gene protein (MIC) on the surface of placenta-derived exosomes.^48^ The interaction with these ligands causes the selective and dose-dependent downregulation of receptor NKG2D, which is present in NK cells, CDC8^+^, and gamma delta T cells, without affecting the lytic pathway through perforin.^48^ Exosomes also have a role in expression of components of the B7 family of ligands including B7-H3, which causes downregulation of T cell activation.^20^ HLA-G5 isoform incorporation within exosomes is another important aspect of exosome involvement, as this class of molecules has been shown to protect fetal tissue from maternal immune cell attack.^20^

More specifically, placental EVs inhibit maternal immunity and promote fetal survival through the expression of specific immunoregulatory molecules.^45,47,48^ For instance, syncytin-1 is present in placental EV cells. In normal pregnancies, syncytin-1 suppresses the production of tumor necrosis factor alpha (TNF-α) and interferon gamma (IFN-γ), inflammatory regulators that have links to early pregnancy loss and PE.^50,51^ In a related manner, maternal EVs can also be proinflammatory, causing a systemic inflammatory state and resulting in pathologic pregnancies. Studies have shown that placental EVs can induce release of proinflammatory cytokines, including TNF-α, macrophage inflammatory protein (MIP)-1α, interleukin (IL)-1α, -6, -8, and -1β from endothelial cells and activate macrophages to release proinflammatory IL-1β.^6,52^ The activation of phagocytic cells, including macrophages and monocytes, is particularly important, as they regulate maternal immune response to maintain a normal pregnancy and also protect against infection.^52,53^ Similarly, monocyte internalization of EVs trigger their migration and the production of IL-1β, IL-6, granulocyte colony stimulating factor (G-CSF), and TNF-α.^53^

Alterations in the levels of inflammatory cytokines in pregnancy result in metabolic disorders such as GDM and PE. The amount of cytokine release is particularly evident in obese pregnant women.^54^ Because of the compounded effect of placental cytokine release and also cytokine release by adipose tissue, the inflammatory pathways are heightened.^55^ Increased body mass index in pregnancy has been shown to be associated with elevated levels of monocyte chemoattractant protein-1 (MCP-1) and TNF-α and increased activation of the p38 mitogen-activated protein kinases (MAPK) and signal transducer and activator of transcription 3 (STAT3) inflammatory pathways.^56^ These proinflammatory states may modify placental function and in turn have fetal effects.

The outcome of aberrant regulation of inflammatory pathways has been established^57-59^; however, the molecules specifically responsible for these effects are still not well understood. The content of EVs reflects the cellular microenvironment and the physiologic response to stresses in the microenvironment and thus may be an important aspect in further understanding the physiologic mechanisms in pregnancy. Current data suggest that exosomes in maternal circulation may contribute to development of a proinflammatory environment by transferring specific cargo (protein and microRNAs [miRNAs]) to target cells.

## EXTRACELLULAR VESICLES IN FETAL CIRCULATION

Once established, maternal-fetal circulation is a bidirectional system that nonselectively allows for the exchange between mother and fetus of nutrients, oxygen, wastes, and cytokines necessary for fetal growth. Because of the bidirectional passage, pathologic changes affecting maternal adaptations may also complicate the fetus. On the other hand, cellular trafficking from the fetal to maternal compartment leads to fetal microchimerism, and an appropriate maternal immune response to this phenomenon is key for the proper maintenance of pregnancy.^60,61^ Furthermore, cell-to-cell signaling from mother to fetus in complicated pregnancies helps protect the fetus via compensatory mechanisms.

Exosomes have been isolated from fetal cord blood in several studies^33,58-60,62,63^; however, Miranda et al examined the contribution of placental exosomes to the total exosome concentration in fetal circulation and found that the concentration of placental exosomes in maternal and fetal circulation is decreased in conditions such as fetal growth restriction and small for gestational age.^64^ Cleys et al studied the umbilical cord blood and serum in pregnant sheep and showed the presence of placental EVs.^65^ A comparison between miRNAs of these vesicles isolated from the umbilical cord blood and maternal serum demonstrated a difference in the miRNA content. Further evaluation by bioinformatics analysis revealed that the miRNA in fetal circulation is important for embryonic development, and in states with abnormal stressors on maternal development, the expression profile of exosomes is also altered.^62^ The proteins that were expressed differently caused different outcomes for regulation of cellular processes, including complement and coagulation cascades, enzyme regulator activity, and extracellular regulation. Identifying placental-specific exosomes in fetal circulation and isolating their specific roles in both healthy and pathologic pregnancies are important areas of research and need further investigation.

## CONCLUSION

EVs represent a mechanism of maternal-fetal interaction during gestation. Exosomes provide a way to identify the function and metabolic state of cell origin through their ability to reflect the microenvironment that they are released from and the metabolic state of their cell of origin. Studies have demonstrated that EVs are released from the placenta into the maternal circulation and have a wide range of functions to regulate immunologic responses to pregnancy and to establish the maternal vascular function. Further understanding of how EVs regulate key events in pregnancy may help elucidate how maternal-fetal communication is established in both normal and pathologic conditions.

## Notes

This article meets the Accreditation Council for Graduate Medical Education and the American Board of Medical Specialties Maintenance of Certification competencies for Patient Care and Medical Knowledge.

## References

[1] Burton GJ, Fowden AL The placenta: a multifaceted, transient organ. Philos Trans R Soc Lond B Biol Sci. 2015 Mar 5;370(1663):20140066. doi: 10.1098/rstb.2014.0066. 25602070 PMC4305167

[2] Burton GJ, Jauniaux E What is the placenta? Am J Obstet Gynecol. 2015 10;213(4 Suppl):S6.e1, S6-S8. doi: 10.1016/j.ajog.2015.07.050. 26428504

[3] Mitchell MD, Peiris HN, Kobayashi M, Placental exosomes in normal and complicated pregnancy. Am J Obstet Gynecol. 2015 10;213(4 Suppl):S173-S181. doi: 10.1016/j.ajog.2015.07.001. 26428497

[4] Tannetta D, Masliukaite I, Vatish M, Redman C, Sargent I Update of syncytiotrophoblast derived extracellular vesicles in normal pregnancy and preeclampsia. J Reprod Immunol. 2017 2;119:98-106. doi: 10.1016/j.jri.2016.08.008. 27613663

[5] Sarker S, Scholz-Romero K, Perez A, Placenta-derived exosomes continuously increase in maternal circulation over the first trimester of pregnancy. J Transl Med. 2014 Aug 8;12:204. doi: 10.1186/1479-5876-12-204. 25104112 PMC4283151

[6] Salomon C, Scholz-Romero K, Sarker S, Gestational diabetes mellitus is associated with changes in the concentration and bioactivity of placenta-derived exosomes in maternal circulation across gestation. Diabetes. 2016 3;65(3):598-609. doi: 10.2337/db15-0966. 26718504

[7] Redman CW, Sargent IL Placental debris, oxidative stress and pre-eclampsia. Placenta. 2000 9;21(7):597-602. doi: 10.1053/plac.2000.0560. 10985960

[8] Adam S, Elfeky O, Kinhal V, Review: fetal-maternal communication via extracellular vesicles - implications for complications of pregnancies. Placenta. 2017 6;54:83-88. doi: 10.1016/j.placenta.2016.12.001. 27939894

[9] Tannetta D, Dragovic R, Alyahyaei Z, Southcombe J Extracellular vesicles and reproduction-promotion of successful pregnancy. Cell Mol Immunol. 2014 11;11(6):548-563. doi: 10.1038/cmi.2014.42. 24954226 PMC4220835

[10] Buca D, Bologna G, D'Amico A, Extracellular vesicles in feto-maternal crosstalk and pregnancy disorders. Int J Mol Sci. 2020 Mar 19;21(6):2120. doi: 10.3390/ijms21062120. PMC7139847 32204473

[11] Nair S, Salomon C Extracellular vesicles as critical mediators of maternal-fetal communication during pregnancy and their potential role in maternal metabolism. Placenta. 2020 Sep 1;98:60-68. doi: 10.1016/j.placenta.2020.06.011. 33039033

[12] Colombo M, Raposo G, Thery C Biogenesis, secretion, and intercellular interactions of exosomes and other extracellular vesicles. Annu Rev Cell Dev Biol. 2014;30:255-289. doi: 10.1146/annurev-cellbio-101512-122326. 25288114

[13] Salomon C, Yee S, Scholz-Romero K, Extravillous trophoblast cells-derived exosomes promote vascular smooth muscle cell migration. Front Pharmacol. 2014 Aug 11;5:175. doi: 10.3389/fphar.2014.00175. 25157233 PMC4128075

[14] Dragovic RA, Southcombe JH, Tannetta DS, Redman CW, Sargent IL Multicolor flow cytometry and nanoparticle tracking analysis of extracellular vesicles in the plasma of normal pregnant and pre-eclamptic women. Biol Reprod. 2013 Dec 26;89(6):151. doi: 10.1095/biolreprod.113.113266. 24227753

[15] Yamamoto S, Niida S, Azuma E, Inflammation-induced endothelial cell-derived extracellular vesicles modulate the cellular status of pericytes. Sci Rep. 2015 Feb 17;5:8505. doi: 10.1038/srep08505. 25687367 PMC4330530

[16] Lee TH, D'Asti E, Magnus N, Al-Nedawi K, Meehan B, Rak J Microvesicles as mediators of intercellular communication in cancer—the emerging science of cellular 'debris'. Semin Immunopathol. 2011 9;33(5):455-467. doi: 10.1007/s00281-011-0250-3. 21318413

[17] Record M Intercellular communication by exosomes in placenta: a possible role in cell fusion? Placenta. 2014 5;35(5):297-302. doi: 10.1016/j.placenta.2014.02.009. 24661568

[18] Nair S, Salomon C Extracellular vesicles and their immunomodulatory functions in pregnancy. Semin Immunopathol. 2018 9;40(5):425-437. doi: 10.1007/s00281-018-0680-2. 29616307

[19] Muralidharan-Chari V, Clancy J, Plou C, ARF6-regulated shedding of tumor cell-derived plasma membrane microvesicles. Curr Biol. 2009 Dec 1;19(22):1875-1885. doi: 10.1016/j.cub.2009.09.059. 19896381 PMC3150487

[20] Kshirsagar SK, Alam SM, Jasti S, Immunomodulatory molecules are released from the first trimester and term placenta via exosomes. Placenta. 2012 12;33(12):982-990. doi: 10.1016/j.placenta.2012.10.005. 23107341 PMC3534832

[21] Orozco AF, Jorgez CJ, Ramos-Perez WD, Placental release of distinct DNA-associated micro-particles into maternal circulation: reflective of gestation time and preeclampsia. Placenta. 2009 10;30(10):891-897. doi: 10.1016/j.placenta.2009.06.012. 19692120 PMC2758063

[22] Atay S, Gercel-Taylor C, Kesimer M, Taylor DD Morphologic and proteomic characterization of exosomes released by cultured extravillous trophoblast cells. Exp Cell Res. 2011 May 1;317(8):1192-1202. doi: 10.1016/j.yexcr.2011.01.014. 21276792

[23] Rice GE, Scholz-Romero K, Sweeney E, The effect of glucose on the release and bioactivity of exosomes from first trimester trophoblast cells. J Clin Endocrinol Metab. 2015 10;100(10):E1280-E1288. doi: 10.1210/jc.2015-2270. 26241326

[24] Salomon C, Kobayashi M, Ashman K, Sobrevia L, Mitchell MD, Rice GE Hypoxia-induced changes in the bioactivity of cytotrophoblast-derived exosomes. PLoS One. 2013 Nov 11;8(11):e79636. doi: 10.1371/journal.pone.0079636. 24244532 PMC3823597

[25] Salomon C, Torres MJ, Kobayashi M, A gestational profile of placental exosomes in maternal plasma and their effects on endothelial cell migration. PLoS One. 2014 Jun 6;9(6):e98667. doi: 10.1371/journal.pone.0098667. 24905832 PMC4048215

[26] Salomon C, Ryan J, Sobrevia L, Exosomal signaling during hypoxia mediates microvascular endothelial cell migration and vasculogenesis. PLoS One. 2013 Jul 8;8(7):e68451. doi: 10.1371/journal.pone.0068451. 23861904 PMC3704530

[27] Lok CA, Van Der Post JA, Sargent IL, Changes in microparticle numbers and cellular origin during pregnancy and preeclampsia. Hypertens Pregnancy. 2008;27(4):344-360. doi: 10.1080/10641950801955733. 19003636

[28] Goswami D, Tannetta DS, Magee LA, Excess syncytiotrophoblast microparticle shedding is a feature of early-onset pre-eclampsia, but not normotensive intrauterine growth restriction. Placenta. 2006 1;27(1):56-61. doi: 10.1016/j.placenta.2004.11.007. 16310038

[29] Nair S, Jayabalan N, Guanzon D, Human placental exosomes in gestational diabetes mellitus carry a specific set of miRNAs associated with skeletal muscle insulin sensitivity, clinical science. Clin Sci (Lond). 2018 Nov 29;132(22):2451-2467. doi: 10.1042/CS20180487. 30254065

[30] Jayabalan N, Lai A, Ormazabal V, Adipose tissue exosomal proteomic profile reveals a role on placenta glucose metabolism in gestational diabetes mellitus. J Clin Endocrinol Metab. 2019 May 1;104(5):1735-1752. doi: 10.1210/jc.2018-01599. 30517676

[31] Jayabalan N, Lai A, Nair S, Quantitative proteomics by SWATH-MS suggest an association between circulating exosomes and maternal metabolic changes in gestational diabetes mellitus. Proteomics. 2019 1;19(1-2):e1800164. doi: 10.1002/pmic.201800164. 30536821

[32] Salomon C, Guanzon D, Scholz-Romero K, Placental exosomes as early biomarker of preeclampsia - potential role of exosomal microRNAs across gestation. J Clin Endocrinol Metab. 2017 Sep 1;102(9):3182-3194. doi: 10.1210/jc.2017-00672. 28531338

[33] Jia F, Li J, Rui C, Comparative proteomic profile of the human umbilical cord blood exosomes between normal and preeclampsia pregnancies with high-resolution mass spectrometry. Cell Physiol Biochem. 2015;36(6):2299-2306. doi: 10.1159/000430193. 26279434

[34] Sabapatha A, Gercel-Taylor C, Taylor DD Specific isolation of placenta-derived exosomes from the circulation of pregnant women and their immunoregulatory consequences. Am J Reprod Immunol. 2006 Nov-Dec;56(5-6):345-355. doi: 10.1111/j.1600-0897.2006.00435.x. 17076679

[35] Luo SS, Ishibashi O, Ishikawa G, Human villous trophoblasts express and secrete placenta-specific microRNAs into maternal circulation via exosomes. Biol Reprod. 2009 10;81(4):717-729. doi: 10.1095/biolreprod.108.075481. 19494253

[36] Pillay P, Maharaj N, Moodley J, Mackraj I Placental exosomes and pre-eclampsia: maternal circulating levels in normal pregnancies and, early and late onset pre-eclamptic pregnancies. Placenta. 2016 10;46:18-25. doi: 10.1016/j.placenta.2016.08.078. 27697217

[37] da Silva Nardi F, Michelon TF, Neumann J, High levels of circulating extracellular vesicles with altered expression and function during pregnancy. Immunobiology. 2016 7;221(7):753-760. doi: 10.1016/j.imbio.2016.03.001. 27005781

[38] Ratajczak J, Mierzejewska K, Borkowska S, Kucia M, Ratajczak MZ Novel evidence that human umbilical cord blood-purified CD133+cells secrete several soluble factors and microvesicles/exosomes that mediate paracrine, pro-angiopoietic effects of these cells – implications for and important role of paracrine effects in stem cell therapies in regenerative medicine. Blood. 2013;122(21):1216. doi: 10.1182/blood.V122.21.1216.1216.

[39] Chen Y, Huang Y, Jiang R, Teng Y Syncytiotrophoblast-derived microparticle shedding in early-onset and late-onset severe pre-eclampsia. Int J Gynaecol Obstet. 2012 12;119(3):234-238. 22986096 10.1016/j.ijgo.2012.07.010

[40] Moro L, Bardaji A, Macete E, Placental microparticles and microRNAs in pregnant women with plasmodium falciparum or HIV infection. PLoS One. 2016 Jan 12;11(1):e0146361. doi: 10.1371/journal.pone.0146361. 26757431 PMC4710532

[41] Lok CAR, Böing AN, Sargent IL, Circulating platelet-derived and placenta-derived microparticles expose Flt-1 in preeclampsia. Reprod Sci. 2008 12;15(10):1002-1010. doi: 10.1177/1933719108324133. 18936439

[42] Li T, Yan Y, Wang B, Exosomes derived from human umbilical cord mesenchymal stem cells alleviate liver fibrosis. Stem Cells Dev. 2013 Mar 15;22(6):845-854. doi: 10.1089/scd.2012.0395. 23002959 PMC3585469

[43] Zhou Y, Xu H, Xu W, Exosomes released by human umbilical cord mesenchymal stem cells protect against cisplatin-induced renal oxidative stress and apoptosis in vivo and in vitro. Stem Cell Res Ther. 2013 Apr 25;4(2):34. doi: 10.1186/scrt194. 23618405 PMC3707035

[44] Mincheva-Nilsson L, Baranov V Placenta-derived exosomes and syncytiotrophoblast microparticles and their role in human reproduction: immune modulation for pregnancy success. Am J Reprod Immunol. 2014 11;72(5):440-457. doi: 10.1111/aji.12311. 25164206

[45] Stenqvist AC, Nagaeva O, Baranov V, Mincheva-Nilsson L Exosomes secreted by human placenta carry functional Fas ligand and TRAIL molecules and convey apoptosis in activated immune cells, suggesting exosome-mediated immune privilege of the fetus. J Immunol. 2013 Dec 1;191(11):5515-5523. doi: 10.4049/jimmunol.1301885. 24184557

[46] Vargas A, Zhou S, Ethier-Chiasson M, Syncytin proteins incorporated in placenta exosomes are important for cell uptake and show variation in abundance in serum exosomes from patients with preeclampsia. FASEB J. 2014 8;28(8):3703-3719. doi: 10.1096/fj.13-239053. 24812088

[47] Germain SJ, Sacks GP, Soorana SR, Sargent IL, Redman CW Systemic inflammatory priming in normal pregnancy and preeclampsia: the role of circulating syncytiotrophoblast microparticles. J Immunol. 2007 May 1;178(9):5949-5956. doi: 10.4049/jimmunol.178.9.5949. 17442979

[48] Hedlund M, Stenqvist AC, Nagaeva O, Human placenta expresses and secretes NKG2D ligands via exosomes that down-modulate the cognate receptor expression: evidence for immunosuppressive function. J Immunol. 2009 Jul 1;183(1):340-351. doi: 10.4049/jimmunol.0803477. 19542445

[49] Southcombe J, Tannetta D, Redman C, Sargent I The immunomodulatory role of syncytiotrophoblast microvesicles. PLoS One. 2011;6(5):e20245. doi: 10.1371/journal.pone.0020245. 21633494 PMC3102084

[50] Than NG, Abdul Rahman O, Magenheim R, Placental protein 13 (galectin-13) has decreased placental expression but increased shedding and maternal serum concentrations in patients presenting with preterm pre-eclampsia and HELLP syndrome. Virchows Arch. 2008 10;453(4):387-400. doi: 10.1007/s00428-008-0658-x. 18791734 PMC2775473

[51] Holder BS, Tower CL, Forbes K, Mulla MJ, Aplin JD, Abrahams VM Immune cell activation by trophoblast-derived microvesicles is mediated by syncytin 1. Immunology. 2012 6;136(2):184-191. doi: 10.1111/j.1365-2567.2012.03568.x. 22348442 PMC3403269

[52] Atay S, Gercel-Taylor C, Taylor DD Human trophoblast-derived exosomal fibronectin induces pro-inflammatory IL-1beta production by macrophages. Am J Reprod Immunol. 2011 10;66(4):259-269. doi: 10.1111/j.1600-0897.2011.00995.x. 21410811

[53] Atay S, Gercel-Taylor C, Suttles J, Mor G, Taylor DD Trophoblast-derived exosomes mediate monocyte recruitment and differentiation. Am J Reprod Immunol. 2011 1;65(1):65-77. doi: 10.1111/j.1600-0897.2010.00880.x. 20560914

[54] Madan JC, Davis JM, Craig WY, Maternal obesity and markers of inflammation in pregnancy. Cytokine. 2009 7;47(1):61-64. doi: 10.1016/j.cyto.2009.05.004. 19505831

[55] Jayabalan N, Nair S, Nuzhat Z, Cross talk between adipose tissue and placenta in obese and gestational diabetes mellitus pregnancies via exosomes. Front Endocrinol (Lausanne). 2017 Sep 27;8:239. doi: 10.3389/fendo.2017.00239. 29021781 PMC5623931

[56] Aye ILMH, Jansson T, Powell TL Interleukin-1β inhibits insulin signaling and prevents insulin-stimulated system A amino acid transport in primary human trophoblasts. Mol Cell Endocrinol. 2013 Dec 5;381(1-2):46-55. doi: 10.1016/j.mce.2013.07.013. 23891856 PMC3795822

[57] Cotechini T, Graham CH Aberrant maternal inflammation as a cause of pregnancy complications: a potential therapeutic target? Placenta. 2015 8;36(8):960-966. doi: 10.1016/j.placenta.2015.05.016. 26094029

[58] Perucci LO, Corrêa MD, Dusse LK, Gomes KB, Sousa LP Resolution of inflammation pathways in preeclampsia—a narrative review. Immunologic Research. Immunol Res. 2017 8;65(4):774-789. doi: 10.1007/s12026-017-8921-3. 28391374

[59] Nguyen-Ngo C, Jayabalan N, Salomon C, Lappas M Molecular pathways disrupted by gestational diabetes mellitus. J Mol Endocrinol. 2019 10;63(3):R51-R72. doi: 10.1530/JME-18-0274. 31416051

[60] Jeanty C, Derderian SC, MacKenzie TC Maternal-fetal cellular trafficking: clinical implications and consequences. Curr Opin Pediatr. 2014 6;26(3):377-382. doi: 10.1097/MOP.0000000000000087. 24759226 PMC4079801

[61] Kallenbach LR, Bianchi DW, Peter I, Stroh H, Johnson KL Maternal background strain influences fetal-maternal trafficking more than maternal immune competence in mice. J Reprod Immunol. 2011 8;90(2):188-194. doi: 10.1016/j.jri.2011.05.004. 21733578 PMC3143711

[62] Campello E, Spiezia L, Radu CM, Circulating microparticles in umbilical cord blood in normal pregnancy and pregnancy with preeclampsia. Thromb Res. 2015 8;136(2):427-431. doi: 10.1016/j.thromres.2015.05.029. 26037284

[63] Panfoli I, Ravera S, Podestà M, Exosomes from human mesenchymal stem cells conduct aerobic metabolism in term and preterm newborn infants. FASEB J. 2016 4;30(4):1416-1424. doi: 10.1096/fj.15-279679. 26655706

[64] Miranda J, Paules C, Nair S, Placental exosomes profile in maternal and fetal circulation in intrauterine growth restriction - liquid biopsies to monitoring fetal growth. Placenta. 2018 4;64:34-43. doi: 10.1016/j.placenta.2018.02.006. 29626979

[65] Cleys ER, Halleran JL, McWhorter E, Identification of microRNAs in exosomes isolated from serum and umbilical cord blood, as well as placentomes of gestational day 90 pregnant sheep. Mol Reprod Dev. 2014 11;81(11):983-993. doi: 10.1002/mrd.22420. 25269776

